# Accelerated Growth of *Corynebacterium glutamicum* by Up-Regulating Stress- Responsive Genes Based on Transcriptome Analysis of a Fast-Doubling Evolved Strain

**DOI:** 10.4014/jmb.2006.06035

**Published:** 2020-07-14

**Authors:** Jihoon Park, SuRin Lee, Min Ju Lee, Kyunghoon Park, Seungki Lee, Jihyun F. Kim, Pil Kim

**Affiliations:** 1 Department of Biotechnology, The Catholic University of Korea, Gyeonggi 14662, Republic of Korea; 2 Department of Systems Biology, Division of Life Sciences, and Institute for Life Science and Biotechnology, Yonsei University, Seoul 03722, Republic of Korea

**Keywords:** *Corynebacterium glutamicum*, adaptive laboratory evolution, rapid reproduction, cellular energy, oxidative stress management, translation process

## Abstract

*Corynebacterium glutamicum*, an important industrial strain, has a relatively slower reproduction rate. To acquire a growth-boosted *C. glutamicum*, a descendant strain was isolated from a continuous culture after 600 generations. The isolated descendant *C. glutamicum*, JH41 strain, was able to double 58% faster (t_d_=1.15 h) than the parental type strain (PT, t_d_=1.82 h). To understand the factors boosting reproduction, the transcriptomes of JH41 and PT strains were compared. The mRNAs involved in respiration and TCA cycle were upregulated. The intracellular ATP of the JH41 strain was 50% greater than the PT strain. The upregulation of NCgl1610 operon (a putative dyp-type heme peroxidase, a putative copper chaperone, and a putative copper importer) that presumed to role in the assembly and redox control of cytochrome *c* oxidase was found in the JH41 transcriptome. Plasmid-driven expression of the operon enabled the PT strain to double 19% faster (t_d_=1.82 h) than its control (t_d_=2.17 h) with 14% greater activity of cytochrome *c* oxidase and 27% greater intracellular ATP under the oxidative stress conditions. Upregulations of genes those might enhance translation fitness were also found in the JH41 transcriptome. Plasmid-driven expressions of NCgl0171 (encoding a cold-shock protein) and NCgl2435 (encoding a putative peptidyl-tRNA hydrolase) enabled the PT to double 22% and 32% faster than its control, respectively (empty vector: t_d_=1.93 h, CspA: t_d_=1.58 h, and Pth: t_d_=1.44 h). Based on the results, the factors boosting growth rate in *C. gluctamicum* were further discussed in the viewpoints of cellular energy state, oxidative stress management, and translation.

## Introduction

The reproduction of microorganisms is defined as the increase in their number while maintaining the same cellular components. The doubling time of these cells varies widely depending on environmental factors and genetic factors inherent in each species. *Vibrio natriegens*, one of the fastest doubling microorganism, has been reported to double in less than 10 min in BHIN medium [1]. *V. natriegens* has been studied widely in order to understand this desirable characteristic of rapid cell division for industrial applications. The following genetic factors have been identified to contribute to rapid reproduction: 1) two chromosomes that can replicate independently and rapidly [2]; 2) a large number of rRNA operons (12 sets) that can perform translation efficiently; 3) localization of genes involved in transcription and translation near the bacterial origin of replication (*oriC*) to facilitate assembly of ribosomes; 4) rich in genes related to respiratory pathways that help in rapid cell growth [3]. Several following studies focus on the ways to leverage this species as a host for molecular biology and biotechnology applications [4, 5].

*Corynebacterium glutamicum* has been a useful industrial strain [6] for the production of biochemicals and recombinant proteins [7]. The doubling time of *C. glutamicum* in a defined CGXII medium without protocatechuate is known to be more than 2 h (corresponding growth rate = 0.34 h^-1^) [8]. Given that *E. coli* doubles every 0.9 h in glucose minimal medium [9], *C. glutamicum* grows much slower, which is disadvantageous for industrial use. To overcome this shortcoming and expand its applicability, adaptive laboratory evolution for accelerating the growth rate of *C. glutamicum* can be considered. Adaptive laboratory evolution has been used to gain insights into microbial metabolism by inducing the accumulation of molecular and phenotypic changes in microbial populations over long periods of culture under specific conditions [10]. Microbial evolution is used to develop a variety of strains to improve growth [11, 12], enhance stress tolerance [13], substrate expansion [14], and to increase small molecule production [15]. With this premise, several studies have attempted to obtain an evolutionary *C. glutamicum* strain with a fast growth rate. Pfeifer *et al.* reported a descendant UB strain of

*C. glutamicum* showing 26% faster growth [11]. Graf *et al*. also reported another descendant *C. glutamicum* strain increased a 58% greater specific growth rate [8].

In this study, a descendant of *C. glutamicum* with improved reproduction was isolated by an adaptive laboratory evolution, and the associated genetic factors were analyzed based on transcriptome profiling.

## Materials and Methods

### Strains and Plasmids

*C. glutamicum* home stock strain KCTC 1445 (Korean Collection for Type Cultures, Daejeon, Korea) was used for the parental type (PT) of the experimental evolution. DNA manipulation followed the method described by Sambrook *et al.* using *E. coli* DH10B (Invitrogen Inc., USA) [16]. The strains and plasmids are listed in Table 1 and the oligonucleotides in Table S1. Target gene-disrupted *C. glutamicum* strains were prepared by double crossover using the pKmobsacB-based plasmids. Homologous arms of the target genes were constructed by connecting two fragments amplified from genomic DNA using overlap extension PCR [17]. Oligonucleotide synthesis and plasmid sequence confirmation were performed at a facility of Bionics Inc. (Korea). *pfu* polymerase was purchased from Solgent Inc. (Korea), and restriction enzymes and T4-ligase from New England Biolabs Inc. (USA).

### Media and Batch Cultures

As for the pre-culture in DNA manipulations, LB (Luria-Bertani) complex medium was used for *E. coli* and of BHI (Brain heart infusion) medium for *C. glutamicum*, *C. glutamicum* was incubated at 30°C, 200 rpm in a shaking incubator, and *E. coli* was grown at 37°C, 220 rpm.

For batch cultures of *C. glutamicum* strains, modified MCGC minimal medium (0.9% glucose, 4 g (NH_4_)_2_SO_4_, 3g KH_2_PO_4_, 6 g Na_2_HPO_4_, 1 g NaCl, 1 g sodium citrate dehydrate, 200 μg biotin, 1 mg thiamine-HCl, 20 mg FeSO_4_•7H_2_O, 0.1 g MgSO_4_•7H_2_O, 2 mg MnSO_4_•H_2_O, 2 mg FeCl_3_, 0.5 μg ZnSO_4_•7H_2_O, 0.2 μg CuCl_2_•2H_2_O, 0.1 μg (NH_4_)_6_Mo_7_O_24_•4H_2_O, 0.2 μg Na_2_B_4_O_7_•10H_2_O, 35 μg CaCl_2_ per liter ) was used [18]. A 500 ml baffled-flask containing 50 ml of the modified MCGC medium was used for batch cultures. For a larger lab-scaled batch culture, 5 L-bioreactor (Liflus GX, Hanil, Korea) containing 2 L of the modified MCGC medium equipped with a dissolved oxygen sensor and a pH sensor was operated at 30°C, agitation of 500 rpm, and aeration of 2 vvm. The inoculation of the cultures in flasks and bioreactor were adjusted to be OD_600_=0.1. Biomass was estimated by measuring optical density (OD) at 600 nm for every hour and converted into g_DCW_/l unit by the coefficient of 0.25. To determine initial growth rate under H_2_O_2_-induced oxidative stress conditions, cells were cultured in 96-well tissue culture plate (SPL Inc., Korea) containing 100 μl of modified MCGC medium. The plate was maintained in a micromixer (VS-101M4, Vision Science Inc., Korea) at 30°C, 13,000 rpm. Optical density was measured using a microplate reader (Bio-Rad Inc., USA) at 600 nm. Initial biomass was adjusted to OD_600_=0.1, and culture was operated until the stationary phase. The exponential growth rate in batch cultures in flasks and 96-well plate was converted from linear regression of log biomass concentrations over each process time. Doubling time (t_d_) were converted from the specific growth rate (μ) by the equation of t_d_ = ln 2 /μ.

### Continuous Culture-Driven Adaptive Laboratory Evolution

Long time continuous culture was performed for the adaptive evolution of the PT strain of *C. glutamicum.* Cells were inoculated into a 250 ml baffled-flask equipped with a screw-cap (Duran GL45, Duran Inc., Germany), stainless steel tubing, PEEK tubing, and 0.2 μm PTFE membrane filter for the inlet and outlet. Culture volume was maintained by feeding of fresh medium from a 20 L-reservoir and by removal of culture broth with the same flow rate of fresh medium feeding rate. The culture volume was measured every 12 h to confirm that the error range within 2% of the set size. The culture was incubated at 30°C, 200 rpm in a shaking incubator. Actively growing PT cells were inoculated (initial OD_600_=0.1) without inlet/outlet feedings until cells reached a mid-log phase. After 12 h of the inoculation, pumps for inlet/outlet feedings were started with dilution rate 0.3 h^-1^. The feeding rate was gradually controlled to increase as long as the all cells were not washed-out during the continuous culture period, which allows only the faster doubling descendant population was remained in the culture vessel. Feeding reservoir was aseptically replaced with the fresh one whenever depleted. The schematic of the continuous culture is represented in Fig. S1.

### Transcriptome Analysis of the Descendant

*C. glutamicum* PT and the evolutionary descendant JH41 strains were cultured in the modified MCGC medium for 5 h, and the cells were harvested to analyze transcriptome at a facility for RNA-seq (Chunlab Inc, Korea). Total RNA was extracted from each sample using NucleoSpin RNA kit (MACHEREY-NAGEL Inc, Germany) following the manufacturer’s instructions. The RNA integrity number (RIN) value of all the isolated RNAs was 10. Single- end 50 bp RNA sequencing was then conducted on an Illumina HiSeq 2500 platform. Normalization was performed using RPKM (reads per kilobase of transcript per million mapped reads), RLE (relative log expression), and TMM (trimmed means of m values) approaches. The method with the lowest coefficient of variation was selected. The EggNOG (evolutionary genealogy of genes: non-supervised orthologous groups) Database was used to cluster genes into functionally related groups, while the KEGG Database was used to analyze metabolic pathways. In addition, pathway enrichment analyses using the KEGG Database were performed to identify DEGs (differentially expressed genes) that exhibited significant changes in expression, with false discovery rate FDR (false discovery rate)-corrected P-values ≤ 0.05 and enrichment with Fisher exact test P-values ≤ 0.05. Visualization of the mapping results, DEG analyses, eggNOG, and KEGG were performed using the CLRNASeq program (ChunLab). Finally, the genes corresponding to the remaining significant pathways were compared by fold-change filtering the quantified gene expression profiles.

### Analyses of Intracellular ATP and Organic Acids

Cells were harvested from batch cultures (1 ml) at 5 h by centrifugation (8,000 ×*g*, 4°C, 5 min) and washed twice with sterile distilled water. The cell suspension (0.8 ml) was mixed with 0.2 g of glass beads (212-300 micron) in a screw-capped tube followed by homogenization for 30 s using a bead beater (Model 607, Biospec Inc., USA). After chilling in an ice-bath for 1 min, homogenization was repeated five more times. Cell debris was removed by centrifugation (8,000 ×*g*, 4°C, 5 min) and the cell lysate was used for measuring intracellular ATP and organic acid concentrations.

ATP concentration was measured using an ENLITEN ATP assay system bioluminescence detection kit (#FF2000, Promega Inc, USA). The intensity of light was detected by a luminometer (GloMax-96 Microplate Luminometer, Promega Inc,) after a luciferase-driven reaction [19]. Organic acids were analyzed by high- performance liquid chromatography (HPLC) [20]. Cell lysates were separated on an ion-exchange column (HPX-io-Rad Inc.,) and a UV detector. Isocratic eluent (0.05% H_2_SO_4_) flowed at 60°C at a rate of 0.6 ml/min. All concentration values represent the statistical mean ± s.d., *n* = 3 biologically independent samples.

### Measurement of Cytochrome *c* Oxidase Activity

Whole-cell cytochrome *c* oxidase activity was estimated by measuring the oxidized form of tetramethyl-*p*-phenylenediamine (TMPD) as described [21, 22]. Cells in late exponential growth phase cultured in 500 ml-flask were harvested by centrifugation (2,600 ×*g*, 4°C, 10 min) and washed twice with 0.9% (w/v) NaCl. Approximately 0.1 mg of the prepared cell was dispersed in a 33 mM potassium phosphate buffer (pH 7.0, 1.4 ml) followed by adding 5 μl of 54 M tetramethyl-*p*-phenylenediamine (TMPD). After reacting for 1 minute at room temperature, absorbance at 520 nm was measured using a spectrophotometer (BioSpectrometer basic, Eppendorf Korea, Korea). The absorbance was converted into enzyme activity (nmole of the oxidized TMPD/mg-biomass in a min) with the extinction coefficient (oxidized TMPD = 6.1 mM^-1^ cm^-1^). The activity assay was repeated at least three times with independent biological repeats.

### Measurement of Oxidative Stress Sensitivity

The oxidative stress sensitivity of strains was compared by agar diffusion test placing an H_2_O_2_-containing disc on the top. Cells in log phase in modified MCGC media were diluted with 1% (w/v) agar solution to an OD_600_ of 1 and poured onto a modified MCGC agar plate. A paper disc (6 mm diameter, Adventec, Japan) was placed onto the bottom agar in the plate and soaked with 10 μl of H_2_O_2_ solution. The plates were incubated at 30°C for 24 h, and the diameter of inhibition zones were measured.

## Results

### Adaptive Laboratory Evolution of *C. glutamicum* PT for Rapid Reproduction

**Fig. 1:**
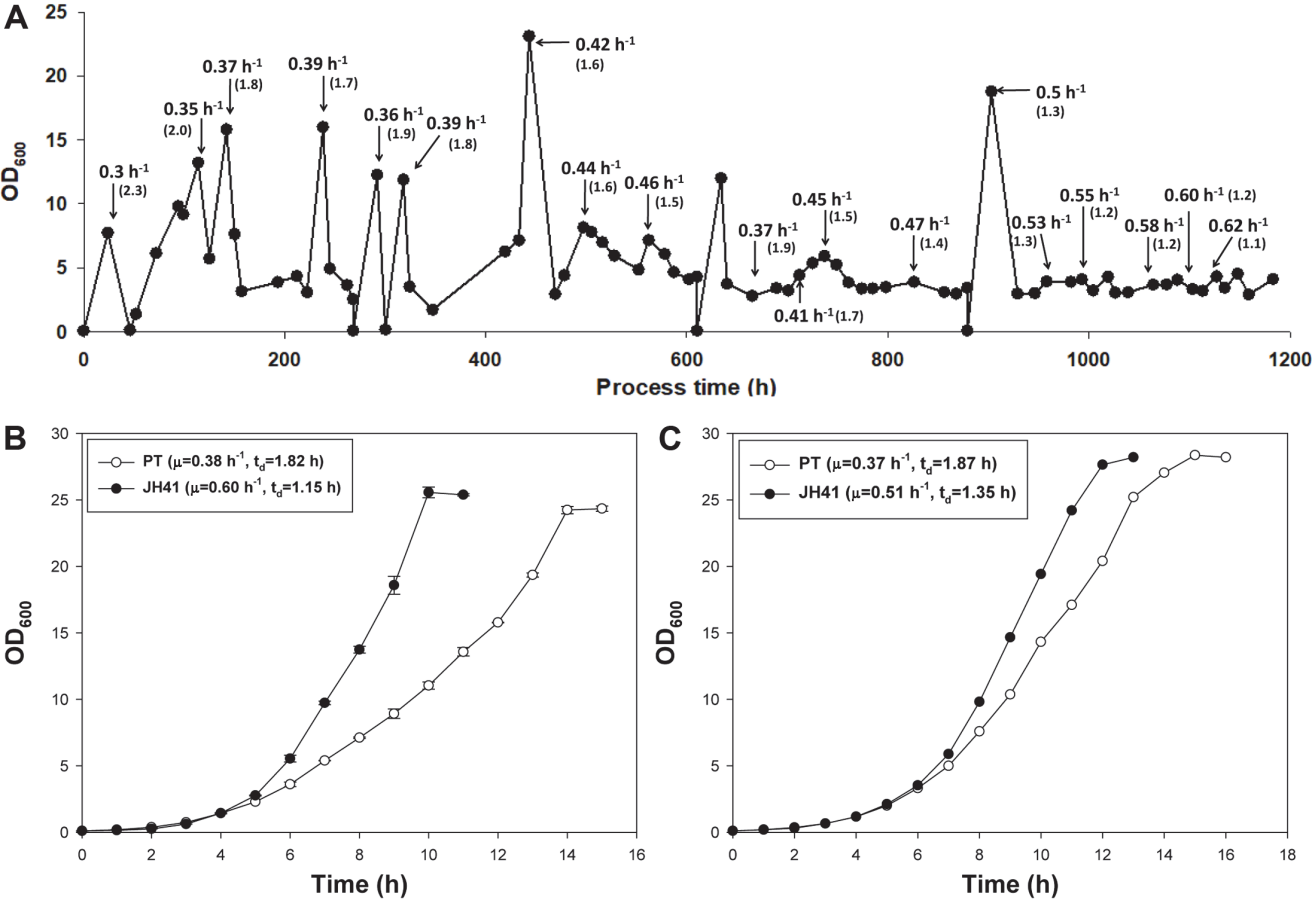
Long-term adaptive laboratory evolution of *C. glutamicum* for rapid reproduction. (**A**) Biomass profile during the adaptive laboratory evolution experiment. Arrows and numbers indicate the dilution rate. Numbers in parenthesis indicate doubling time (h). (**B**) Batch culture growth profiles of PT and JH41 strains. Cultures were in a 500 ml baffled flask containing 50 mL of modified MCGC medium. (**C**) Fermentation growth profiles of PT and JH41 strains. Cultures were in a 5 L fermenter containing 2 L of modified MCGC medium. Each data point represents the mean ± s.d.; *n* = 3 biologically independent samples.

In order to select a rapidly reproducing descendant, a continuous culture of the *C. glutamicum* PT was maintained for 50 days with a gradual increase in feed rate. The reaction scale was reduced to a 250 ml baffled flask scale to minimize the use of medium during long-term continuous culture. The culture volume was maintained at 41 ml during the operation by fixing the location of the outlet tube (Fig. S1). The initial dilution rate was set to 0.3 h^-1^, which was the maximum value at which cells were not washed out in the given conditions. Whenever th optical density (OD) of the outlet was steady for 2 h, the dilution rate was increased in 2-5% higher than the previous flow rate by adjusting the peristaltic pumps. The OD of the culture showed a tendency of instant reduction whenever the pump speed was raised and then began to increase within 24 h until 600 h in process time (ca. 300 generations), indicating that the fast-reproducing descendant cells populated within 24 h (Fig. 1A). The time for recovery of OD after upward adjustment, however, was gradually extended after 600 h of continuous culture. This phenomenon was witnessed in another long-term evolution experiments of *E. coli* [23], where the relative adaptability of the evolved bacteria had increased dramatically over the first few hundreds of generations. In 1,200 h of operation (ca. 600 generations), the dilution rate of the culture was found to be 0.62 h^-1^ (corresponding to t_d_=1.12 h).

The final evolved culture broth was spread on MCGC agar plates, and four largest colonies were selected to transferred to 500 ml baffled flasks containing 50 ml of modified MCGC medium. Among the four descendant liquid cultures, the fastest growing descendant strain (named JH41) showed t_d_=1.15 h, which exhibited a 58% faster growth rate than the ancestral PT strain (t_d_=1.82 h) in 500 ml baffled-flask conditions (Fig. 1B). The faster reproduction of JH41 was confirmed in a 5 L bioreactor containing 2 L medium. The JH41 strain in the 2 L-culture showed a 38% faster growth rate compared to the PT strain in the same condition (PT t_d_=1.87 h and JH41 t_d_=1.35 h) (Fig. 1C). Meanwhile, the growth rates in the 5 L bioreactor scale was generally lower (2-17%) than those in the 500 ml baffled-flask scale, which may be explained by the differences in the oxygen transfer rates arising from differences in aeration and agitation.

### Transcriptome Analysis of JH41

To determine the molecular factors contributing to rapid reproduction, the transcriptome profile of the descendant JH41 strain was compared with that of PT strain by RNA-sequencing. Total RNAs were isolated from the JH41 and PT cells in the same early-log phase (5 h). The transcriptome of JH41 strain resulted in 1315 upregulations and 1318 downregulations compared with the transcriptome of PT strain. The transcriptome data were deposited to NCBI with accession code of PRJNA556334, and the profile of the noticeable genes are categorized in Table 2.

A noticeable change was observed in the gene clusters involved in the respiratory chain and the TCA cycle, most of which were found to be upregulated (Table 2; board A, and B). Meanwhile, the gene cluster of ATP synthase was downregulated (Table 2; board C). In addition, the mRNA levels of NCgl1610 operon comprising three putative genes (encoding a putative copper importer [NCgl1610], a putative chaperone [NCgl1609] and a putative heme peroxidase [NCgl1608]), were more than three times higher in the JH41 strain (Table 2; board D), where the operon was hypothesized to role in the redox modification of copper ion during the assembly of cytochrome *c* oxidase complex (Fig. S2) [24]. The transcript levels of genes encoding cold shock protein (NCgl0171) and a hypothetical protein (NCgl2435) were also increased by 3.9 and 3.4 times, respectively (Table 2; board E), where the hypothetical protein was predicted a putative peptidyl-tRNA hydrolase based on SWISS modeling and CD- search (Fig. S3). Upregulations of genes involving leucine, isoleucine, valine biosynthesis were also noticeable in the JH41 strain (Table S2).

### Intracellular ATP and Organic Acids Concentrations of JH41

The upregulations in the respiration and TCA genes along with the downregulations in the components of F_1_F_0_-ATP synthase (Table 2; board A, B, C) triggered the authors to wonder the levels of intracellular ATP and organic acids in JH41 strain at the same state of the transcriptome variation (Table 3). The ATP level of the PT strain in the log phase was 1.13 ± 0.15 μmole/g_DCW_ while that of JH41 strain was 1.69 ± 0.14 μmole/g_DCW_. Therefore the 50% greater ATP level of JH41 was agreed with the upregulations in the respiration genes. The downregulations of the components of F_1_F_0_-ATP synthase was thought to be a consequent result triggered from the cellular high ATP state. The levels of organic acids resulted that JH41 strain harbored 5-times less pyruvate, 2-times less citrate, 9- times more fumarate, and similar lactate compared with those of PT strain. The actual changes in organic acids levels in JH41 agreed with the transcriptome profiles in Table 2 considering the downregulation of *gltA* (citrate synthase) would reduce intracellular citrate, the upregulation of *pck* (PEP carboxykinase) would reduce intracellular pyruvate, the upregulations of *sdhAB, sdhC, sdhD* (succinate: menaquinone oxidoreductase complex) would increase intracellular fumarate, and the upregulations of leucine, isoleucine, valine biosynthesis (Table S2) would enhance the consumption of pyruvate.

Therefore, the authors were able to assume the higher intracellular ATP and the modified TCA cycle beneficial for more building block synthesis would have contributed to the faster doubling of JH41 strain.

### Counteractions on Oxidative Stress-induced Growth Retardation

**Fig. 2:**
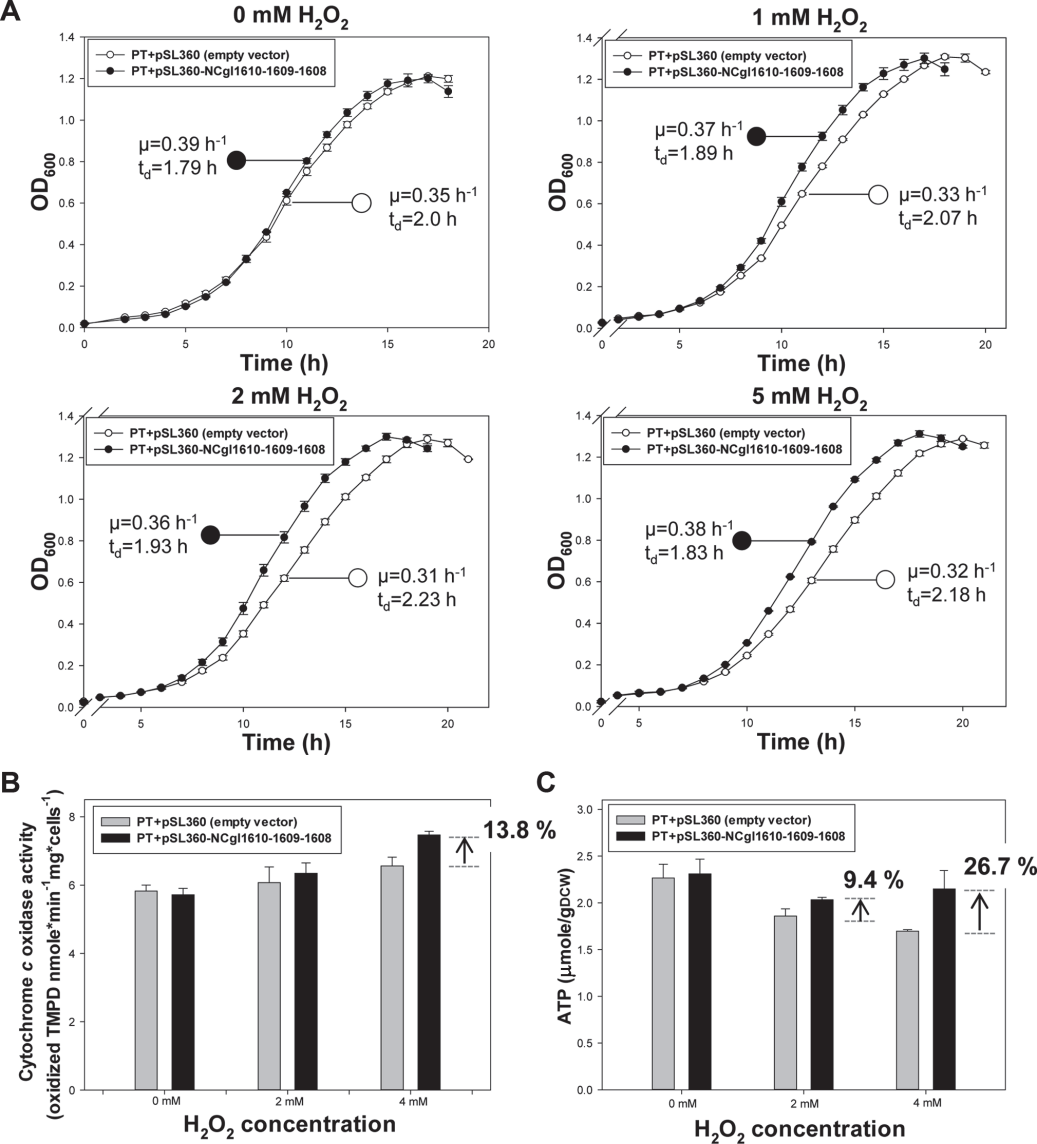
Effect of constitutive expression of NCgl1610 operon on reproduction rate, cytochrome *c* oxidase activity, and ATP production. (**A**) The reproduction rate of *C. glutamicum* strains harboring pSL360 (empty vector) and pSL360-NCgl1610 operon under various H_2_O_2_ concentrations. Each data point is the mean ± s.d.; *n* = 3 biologically independent samples. (**B**) Bar graph showing cytochrome *c* activity and (**C**) intracellular ATP concentration of *C. glutamicum* strains harboring pSL360 (empty vector) and pSL360-NCgl1610 operon under various H_2_O_2_ concentrations. All strains were cultured in modified MCGC media and used for subsequent analyses. TMPD-oxidizing activity was measured when the cells reached the late-log phase and was considered as a read-out of cytochrome *c* oxidase activity. Intracellular ATP concentration was measured when the cells reached mid-log phase as per the luciferin-luciferase reaction. Bar heights and error bars show statistical means ± s.d.; *n* = 3 biologically independent samples.

It was predicted that the greater energy generation of JH41 would lead to simultaneous oxidative stress, and the rapid reproduction would, therefore, require corresponding antioxidation processes. In line, the transcriptome data of JH41 showed a 0.87-fold downregulation of the OxyR gene, which is a representative transcriptional regulator of antioxidant mechanisms. The transcriptome of JH41 also showed the upregulations of a putative heme peroxidase (NCgl1608, 5.16-fold upregulation), a putative copper chaperone (NCgl1609, 4.02-fold upregulation) and a putative copper importer (NCgl1610, 3.47-fold upregulation), that composed a single NCgl1610 operon (Table 2; board D). The putative copper chaperone and the putative copper importer have been reported to be upregulated under non-standard copper concentration and H_2_O_2_-induced oxidative stress conditions [21, 25]. However, the single deletion of putative heme peroxidase or copper chaperone did not show any growth defect, and their functions are still unclear. To confirm if the putative heme peroxidase upregulation contributed to the rapid reproduction in JH41, the operon containing the three genes were constitutively expressed from a plasmid in the PT strain (PT + pSL360-NCgl1608-1609-1610) under various redox conditions, and the growths of the strains were profiled (Fig. 2A). The growth of the PT expressing the operon showed a slightly faster growth pattern compared to that of the control. Interestingly, the effect of operon expression on growth tended to amplify as the H_2_O_2_ concentrations were increased, resulting in up to 19.4% faster growth rate in 5 mM H_2_O_2_ conditions. This result indicated that upregulation of the operon contributed to the rapid reproduction, especially under higher redox stress conditions.

**Fig. 3:**
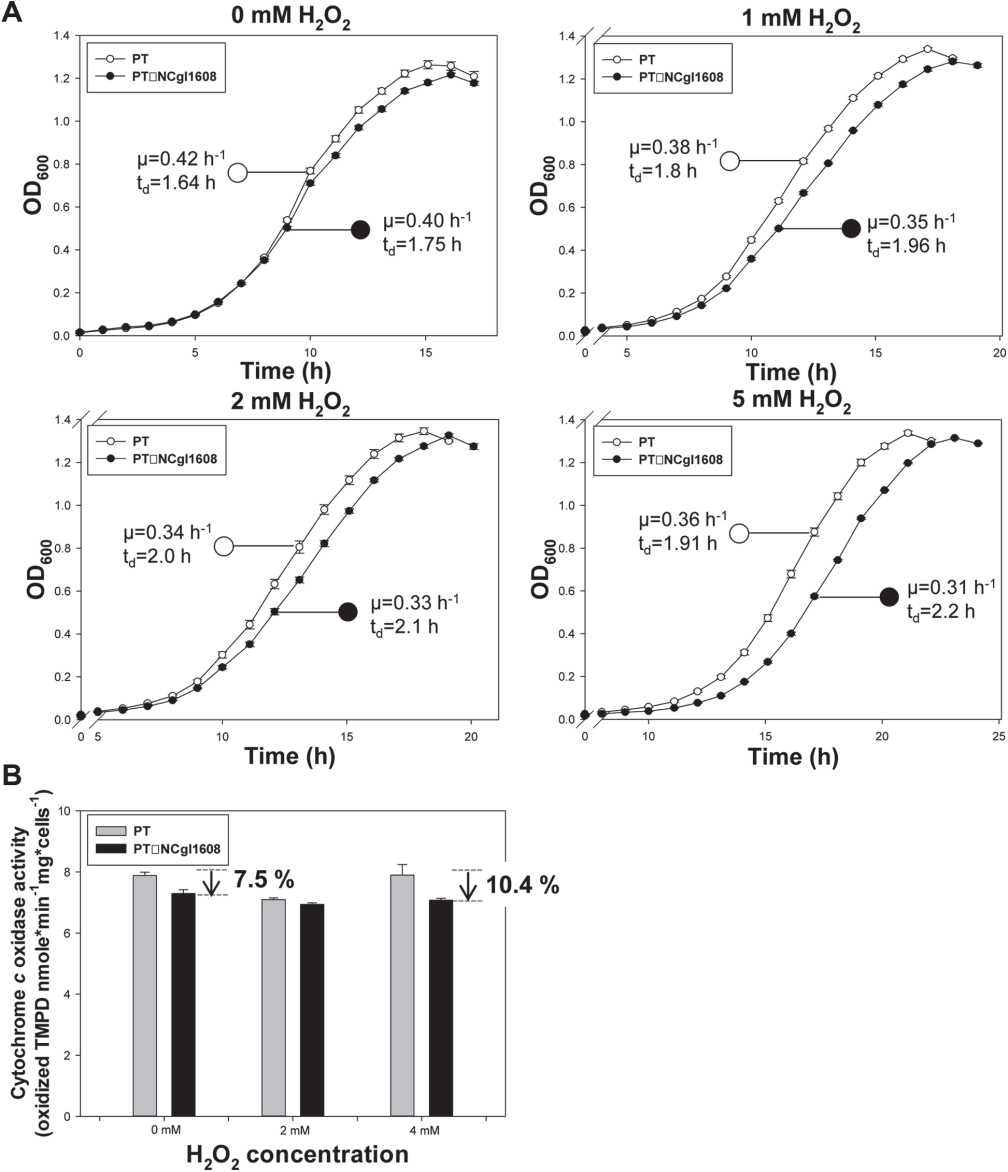
Effect of NCgl1608 (putative heme peroxidase) deletion on reproduction rate, cytochrome *c* oxidase activity. (**A**) The reproduction rate of *C. glutamicum* strains PT and PT ΔNCgl1608 under various H_2_O_2_ concentrations. Each data point is the mean ± s.d.; *n* = 3 biologically independent samples. (**B**) Bar graph showing cytochrome *c* activity of PT and PT ΔNCgl1608 under various H_2_O_2_ concentrations. All strains were cultured in modified MCGC media and used for subsequent analyses. TMPD-oxidizing activity was measured when the cells reached the late-log phase and was considered as a read-out of cytochrome *c* oxidase activity. Bar heights and error bars show statistical means ± s.d.; *n* = 3 biologically independent samples.

**Fig. 4:**
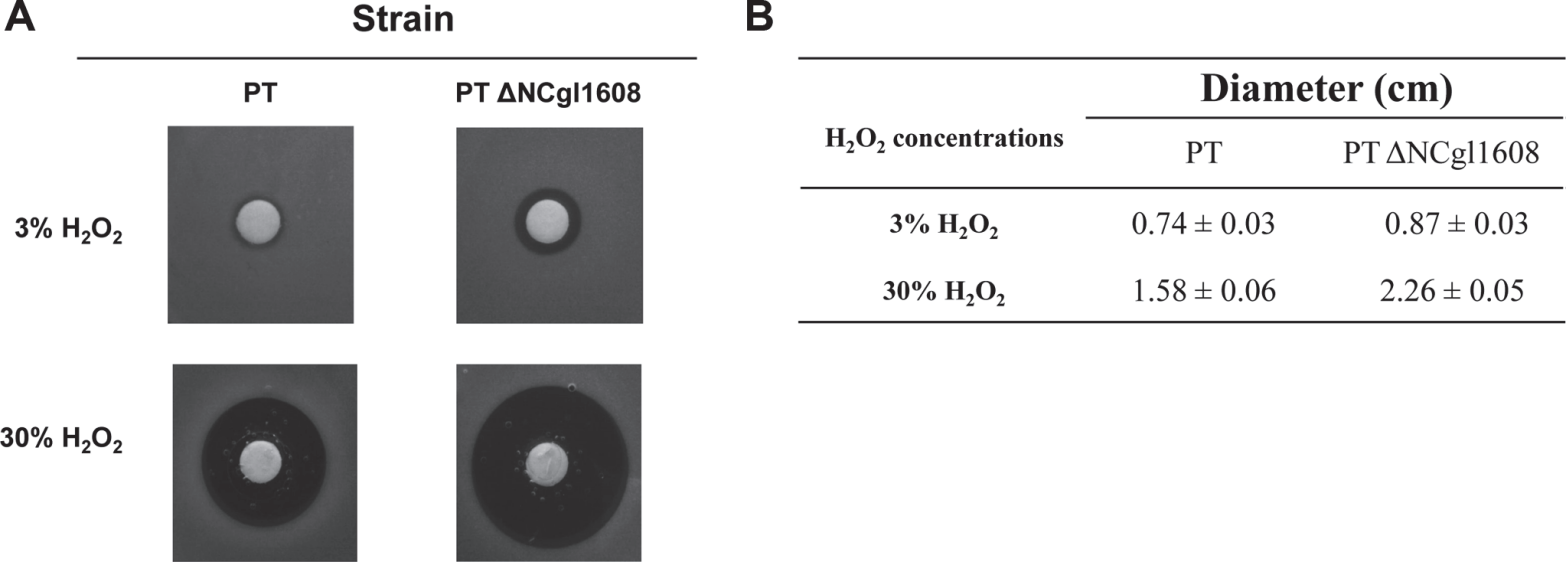
Effect of NCgl1608 (putative heme peroxidase) deletion on H_2_O_2_ sensitivity. (**A**) The oxidative stress sensitivity of strains was compared by agar diffusion test placing an H_2_O_2_-containing disc on the top and (**B**) the diameter of inhibition zones. Values represent the statistical mean ± s.d.; *n* = 3 biologically independent samples.

Referring to the study of Petrus *et al*. [26], we hypothesized that the heme peroxidase might provide a copper element in proper redox state coupled with the copper chaperone and the copper importer leading the appropriate assembly of the electron transfer chain cytochrome *c* (*aa*_3_) complexes, harboring 2 iron and 2 copper elements (Fig. S2). To verify the hypothesis, the cytochrome *c* oxidase activities and intracellular ATP concentrations were measured in the PT strain expressing the three concerned genes (Figs. 2B and Cytochrome *c* oxidase activity and intracellular ATP concentration were congruently found to be increased by 13.8% and 26.7% at 4 mM H_2_O_2_, respectively by the expression of the three genes. On the other hand, a mutant strain disrupted the putative heme peroxidase (PT ΔNCgl1608) showed growth retardation with increasing H_2_O_2_ concentration, and its specific growth rate was found to reduce by up to 12.9% (Fig. 3A). The sensitivity of PT ΔNCgl1608 to oxidative stress was confirmed by agar diffusion assay at 3% and 30% H_2_O_2_ concentrations (Fig. 4). The activity of cytochrome *c* oxidase complex in the PT ΔNCgl1608 strain was also decreased by 10.4% at 4 mM H_2_O_2_ (Fig. 3B), suggesting that the putative heme peroxidase contributes to the activation of cytochrome *c* oxidase. Therefore, the authors suggest that the upregulation of NCgl1610 operon is beneficial for rapid reproduction, by providing proper redox state at the surface of cell and thereby contributing to the assembly of an active cytochrome *c* oxidase complex (Fig. S2).

### Increasing Refreshing Translation Fitness

Transcriptome analysis also showed that mRNAs of NCgl0171 (encoding cold shock protein) and NCgl2435 (encoding a hypothetical protein) were increased by 3.9- and 3.4-times, respectively, in the evolved descendant JH41 strain (Table 2; board E). The cold shock protein was known to act as an mRNA-chaperone by mitigating the secondary structure of mRNA induced at low temperatures, thereby effectuating translation by ribosomes [27]. On the other hand, the hypothetical protein was likely to be a peptidyl-tRNA hydrolase based on SWISS-model sequence homology and CD-search (Fig. S3). The peptidyl-tRNA hydrolase in *E. coli* is known as one of the rescue factors that allows the recycling of stalled ribosomes [28]. Therefore, we predicted that refreshing translation process by linearizing mRNAs and by recycling the stalled ribosome’s subunits might have contribute to the rapid reproduction observed in JH41.

**Fig. 5:**
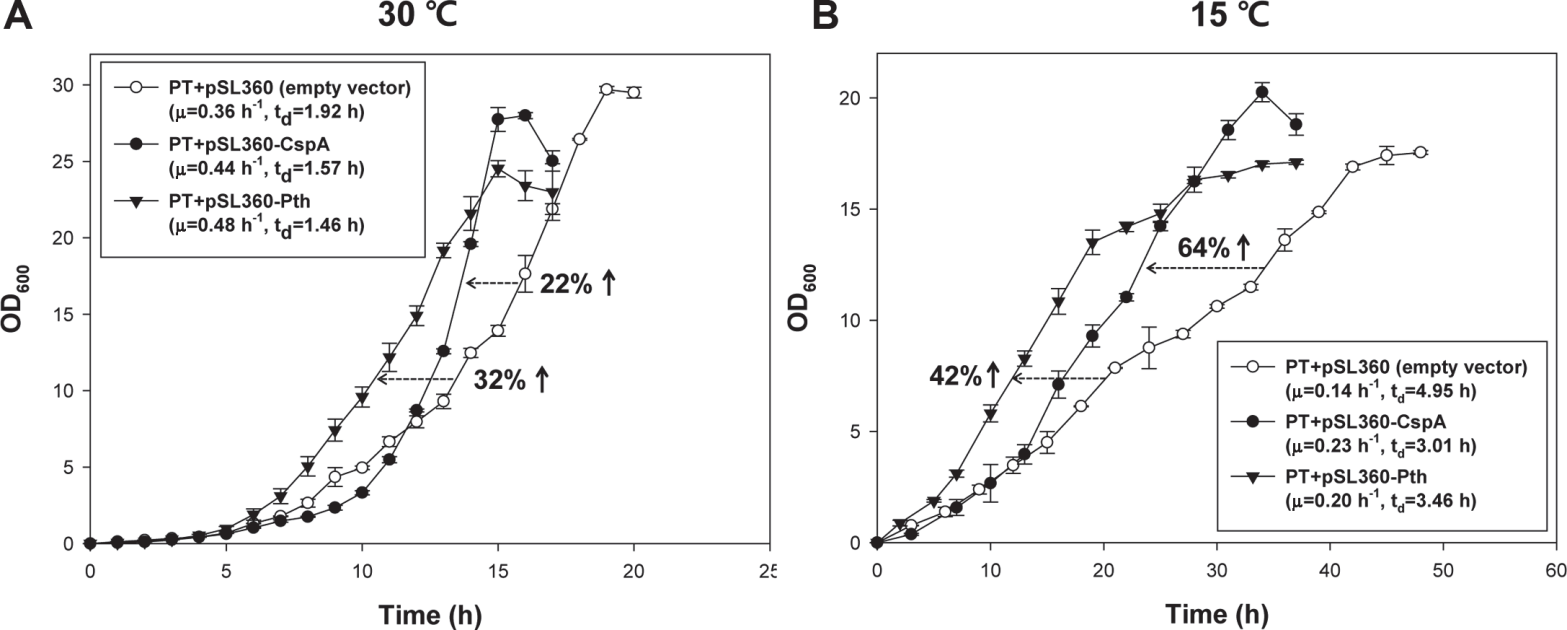
Effects of translation refreshing genes (CspA, putative Pth) on the reproduction rate. (**A**) Growth curve of *C. glutamicum* PT strain expressing CspA and Pth cultured at 30°C; (**B**) growth curve cultured at 15°C. All strains were cultured in a 500 ml baffled flask containing 50 ml of modified MCGC medium. Each data point is the mean s.d.; *n* = 3 biologically independent samples.

To confirm the prediction, the cold shock protein and hypothetical peptidyl-tRNA hydrolase were artificially expressed in the PT strain (Fig. 5). The PT strain expressing the refreshing genes showed a faster growth than the control strain (22% increase upon cold shock protein expression, 32% increase upon hypothetical peptidyl-tRNA hydrolase) at 30°C. Further, the artificial expression of cold shock protein and the hypothetical peptidyl-tRNA hydrolase enabled the PT strain to grow 64% and 42% faster at a low temperature (15°C), which would offer obvious translation interferences because of the cold shock-induced increase in mRNA secondary structures. Therefore, the authors conclude that enhanced translation fitness is another factor contributing to the enhanced reproduction rate in JH41 strain. Consistent with these results, we found that overexpression of CspA and Pth of *E. coli* at low temperature (25°C) also increased their growth rate (44.5% increase in CspA, 25.5% increase in Pth) (data not shown).

## Discussion

A rapidly reproducing descendant of *C. glutamicum* was obtained through adaptive laboratory evolution in continuous culture in 600 generations (Fig. 1A). The transcriptome analysis of the descendant strain led to a set of genetic factors that might govern the rapid reproduction ability of JH41, including I) genes involved in cellular energy biosynthesis such as respiration proteins and TCA cycle components; II) genes involved in antioxidation capacity coupled with copper element incorporation in cytochrome *c* oxidase complex, further leading to the activation of respiration; III) genes involved in refreshing translation fitness. The moderate cooperation of the above factors enabled the JH41 to grow 58% faster than its parental strain (t_d[JH41]_=1.15 h VS. t_d[PT]_=1.82 h).

Maitra *et al*. have suggested a model representing greater ATP harboring cells enhance its growth rate [29]. The coupling of the upregulations of respiration components and succinate: menaquinone oxidoreductase complex (*sdhA, sdhB, sdhCD*) in Table 2 would have increased the reducing power in the JH41 strain, leading the increase of cellular ATP. However, the higher respiration process would concomitantly increase oxidative stress. The downregulation of the anti-oxidative stress regulator *oxyR* (mRNA ratio: 0.8-fold), the downregulations of genes for F_0_F_1_-ATP synthase, and the upregulations of catalase and peroxidase (mRNA ratio: 4.4- and 5.1-fold, respectively) would have represented the greater oxidative stress derived from the greater cellular respiration in the JH41 strain.

In addition, the essential trace metals such as copper, manganese, and zinc are generally related to the activity of various enzymes as well as to intracellular oxidative stress [22, 30]. This is because the exposure of free ions to cytoplasm can cause cell damage through random redox reactions. The NCgl1610 operon of *C. glutamicum* is expected to contribute to the integration of copper into cytochrome *c* oxidase, as for the EfeUOB operon of *Bacillus subtilis* and the SLI4214 operon of *Streptomyces lividans* [26, 31]. The mechanism of the interaction of heme peroxidase coupled with the copper chaperone and the copper importer, with cytochrome *c* oxidase, is still unclear. However, we suggested that the heme peroxidase may switch copper oxidation state while decomposing H_2_O_2_, and the switching charge of the copper ions may increase the affinity for copper chaperones, thus leading to the trafficking of copper to the copper center in cytochrome *c* oxidase (Fig. S2) [24].

The artificial expressions of mRNA-straightening CspA and stalling ribosome-rescuing putative Pth have shortened the doubling time of *C. glutamicum* (Fig. 5). Considering the speed of peptide elongation in a ribosome is correlated to the growth rate [29], both the increased activities of CspA and the putative Pth would have contributed to the translation speed in the *C. glutamicum* by refreshing the translation fitness, which directly coupled with the growth rate. Microbial cells harboring high ATP were reported to produce more recombinant proteins [19, 32], JH41 would also provide a suitable host for the production of recombinant proteins. In this respect, our discovery offers essential cues for the construction of an efficient cell factory and expands the biological knowledge base.

Further studies on the genomic changes of JH41 strain, which have caused the transcriptional variations, would unveil the more detailed information for the rapid reproduction of *C. glutamicum*.

## Supplemental Materials



Supplementary data for this paper are available on-line only at http://jmb.or.kr.

## Figures and Tables

**Table 1:** Strains and plasmids used in this study.

Strains and plasmids	Description	Reference
Strains		
*E. coli* DH10B	F^–^ *mcrA* Δ(*mrr-hsd*RMS-*mcrBC*) Φ80*lacZ*ΔM15 Δ*lacX74 recA1* *endA1 araD139* Δ(*ara leu*)7697 *galU galK rpsL nupG* λ^–^	Invitrogen
Parental type (PT)	Home stock of *C. glutamicum* wild-type, Biotin auxotroph, derived from KCTC No. 1445	[33]
JH41	A descendant of *C. glutamicum* PT, fast reproduction	This study
PT ΔNCgl1608	PT derivative, ΔNCgl1608 encoding a putative heme peroxidase (0.8 kb-deletion)	This study
Plasmids		
pSL360	*E. coli* and *C. glutamicum* shuttle vector, Km^R^	[34]
pK19mobsacB	Suicide vector for double recombination, Km^R^, *sacB* of *Bacillus subtilis*	[35]
pSL360-CspA	pSL360 harboring cold shock protein, CspA (NCgl0171) of *C. glutamicum*	This study
pSL360-Pth	pSL360 harboring putative peptidyl tRNA hydrolase, Pth (NCgl2435) of *C. glutamicum*	This study
pSL360-NCgl1608-1609-1610	pSL360 harboring NCgl1610 operon (putative copper importer, putative copper chaperone, putative heme peroxidase) of *C. glutamicum*	This study
pK19mobsacB-ΔNCgl1608	pK19mobsacB harboring homologous arm for deletion of putative heme peroxidase (NCgl1608)	This study

**Table 2:** mRNA fold-change of genes in the respiratory chain, TCA cycle, F_1_F_0_-ATP synthase, and notable genes in the fast-doubling JH41 strain.

NCgl No.	Gene name	Function of gene product	mRNA ratio^a)^
A. Respiratory chain components	
NCgl0359	*sdhC*	Succinate dehydrogenase, cytochrome *b* subunit	2.59
NCgl0360	*sdhA*	Succinate dehydrogenase, flavoprotein	4.22
Ncgl0361	*sdhB*	Succinate dehydrogenase, iron-sulfur protein subunit	5.72
Ncgl1103	*cydB*	Cytochrome *bd* oxidase, subunit II	2.69
Ncgl1104	*cydA*	Cytochrome *bd* oxidase, subunit I	2.65
NCgl1409	*ndh*	NADH dehydrogenase, non-proton pumping	1.12
Ncgl1926	*mqo*	Malate:quinone oxidoreductase	5.18
Ncgl2109	*qcrB*	Cytochrome *bc*_1_ complex, cytochrome b subunit	2.82
Ncgl2110	*qcrA*	Cytochrome *bc*_1_ complex, Rieske iron-sulfur protein	2.24
Ncgl2111	*qcrC*	Cytochrome *bc*_1_ complex, cytochrome *c*_1_ subunit	1.96
Ncgl2112	*ctaE*	Cytochrome *aa*_3_ oxidase, subunit III	1.42
Ncgl2114	*ctaF*	Cytochrome *aa*_3_ oxidase, subunit IV	1.76
Ncgl2115	*ctaC*	Cytochrome *aa*_3_ oxidase, subunit II	1.26
NCgl2297	*mdh*	NAD-dependent Malate dehydrogenase	1.21
Ncgl2437	*ctaD*	Cytochrome *aa*_3_ oxidase, subunit I	1.43
NCgl2810	*ldh*	NAD-dependent Lactate dehydrogenase	1.39
Ncgl2817	*lldD*	L-Lactate dehydrogenase (using MQ as acceptor)	0.96
B. TCA cycle
NCgl0355	*lpd*	Pyruvate dehydrogenase complex, LPD subunit	1.26
NCgl0359	*sdhCD*	Succinate: menaquinone oxidoreductase	2.59
NCgl0360	*sdhA*	Succinate: menaquinone oxidoreductase	4.22
NCgl0361	*sdhB*	Succinate: menaquinone oxidoreductase	5.72
NCgl0634	*icd*	Isocitrate dehydrogenase	0.95
NCgl0659	*pyc*	Pyruvate carboxylase	1.15
NCgl0795	*gltA*	Citrate synthase	0.73
NCgl0967	*fum*	Fumarase	1.44
NCgl1084	*odhA(kgd)*	OGDHC (oxoglutarate dehydrogenase complex) E1o subunit	0.99
NCgl1482	*acn*	Aconitase	3.95
NCgl1523	*ppc*	PEP Carboxylase	0.72
NCgl1926	*mqo*	Malate: quinone oxidoreductase	5.18
NCgl2008	*pyk*	Pyruvate kinase	0.90
Ncgl2126	*sucB(aceF)*	OGDHC E2o subunit	1.47
NCgl2476	*sucD*	OGDHC E2o subunit	5.57
NCgl2477	*sucC*	Succinyl-CoA synthetase	3.00
NCgl2765	*pck*	PEP carboxykinase	1.96
C. F_1_F_0_-ATP synthase
Ncgl1159	*atpB*	α-Subunit of F_0_ part	0.27
Ncgl1160	*atpE*	γ-Subunit of F_0_ part	0.45
Ncgl1161	*atpF*	β-Subunit of F_0_ part	0.44
Ncgl1162	*atpH*	δ-Subunit of F_1_ part	0.46
Ncgl1163	*atpA*	α-Subunit of F_1_ part	0.50
Ncgl1164	*atpG*	γ-Subunit of F_1_ part	0.54
Ncgl1165	*atpD*	β-Subunit of F_1_ part	0.60
Ncgl1166	*atpC*	ε-Subunit of F_1_ part	0.68
D. Other upregulated genes: NCgl1610 operon
NCgl1610	Putative	Putative copper importer (3^rd^ gene of NCgl1610 operon)	3.47
NCgl1609	Putative	Putative copper chaperone (2^nd^ gene of NCgl1610 operon)	4.02
NCgl1608	Putative	Putative dyp-type heme peroxidase (1^st^ gene of NCgl1610 operon)	5.16
E. Other upregulated genes: translation refreshing genes
NCgl0171	*cspA*	Cold-shock protein, denature wrong dsRNA	3.90
NCgl2435	Putative	Hypothetical protein (presumed peptidyl-tRNA hydrolase)	3.35

^a)^mRNA ratios of JH41/PT of the selected genes. Full transcriptome data is available at NCBI with access code of PRJNA556334.

**Table 3:** Intracellular concentrations of ATP and organic acids in JH41.

Concentrations^*^	PT	JH41
ATP (μmole/g_DCW_)	1.13 ± 0.15	1.69 ± 0.14
Organic acids		
Pyruvate (mmole/g_DCW_)	0.24 ± 0.02	0.04 ± 0.01
Citrate (mmole/g_DCW_)	0.23 ± 0.02	0.12 ± 0.01
Fumarate (mmole/g_DCW_)	0.47 ± 0.03	4.02 ± 0.01
Lactate (mmole/g_DCW_)	1.67 ± 0.02	1.70 ± 0.07

^*^All components were measured at 5 h after inoculation, the same time for the transcriptome variation.
