# Supplementary material for: Accelerated Growth of Corynebacterium glutamicum by Up-Regulating Stress- Responsive Genes Based on Transcriptome Analysis of a Fast-Doubling Evolved Strain
Source: J Microbiol Biotechnol. 2020 Jul 14;30(9):1420–9. doi: 10.4014/jmb.2006.06035 (PMC9728273; doi:10.4014/jmb.2006.06035)
Supplement: Supplementary file 1 [file JMB-30-9-1420-supple.pdf]

J. Microbiol. Biotechnol.
https://doi.org/10.4014/jmb.2006.06035

Supplementary Material

(L 95)

Table S1. Oligonucleotides used in this study.

Oligonucleotide	Sequence (5'-3')	Restriction site	Target gene
P1	GTC GAC AGG AGG AAA ATG GCA CAG GGT ACT	SalI	CspA (pSL360-CspA)
P2	CAT ATG TTA GAG AGC ACG AAC CTG C	NdeI	
P3	GTC GAC AGG AGG AAA ATG CAG ATC GCG TTG	SalI	Pth (pSL360-Pth)
P4	CAT ATG TTA TGG AAT TGC AAC AAC C	NdeI	
P5	GAT ATC GTG GCT TCA GAG AAG AAT CT	EcoRV	NCgl1610 (pSL360-putative copper importer)
P6	ACT AGT TTA TAG AAG GAG TCC GGC	SpeI	NCgl1608 (pSL360-putative heme peroxidase)
P7	TCT AGA ATG GTT TCC CGT AGG	XbaI	Homologous arms flanking the 809 bp deletion fragment inside NCgl1608 (pK19mobsacB-ΔNCgl1608)
P8	TGA ATC TGG TTG CGT GAA CTG TCC AAT GCT GGT TT	-	
P9	GAA CTG TCC AAT GCT GG	-	
P10	AGC ATT GGA CAG TTC ACG CAA CCA GAT TCA AAT TC	-	
P11	ACG CAA CCA GAT TCA AA	-	
P12	CCC GGG GGT TAT AGA AGG AGT CCG	XmaI	

(L 143)

Figure S1. Schematic diagram of the chemostat used for long-term adaptive laboratory evolution.

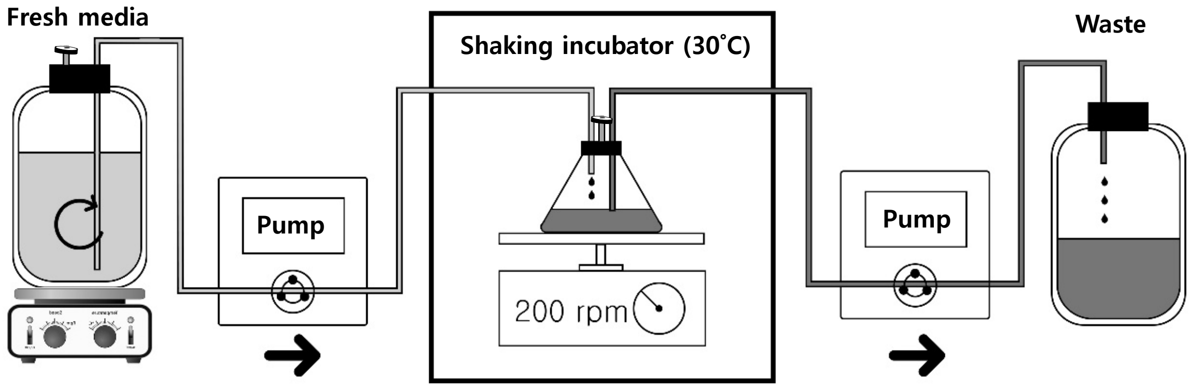

(L 249)

Figure S2. Proposed model for the role of NCgl1610 operon in copper trafficking to cytochrome *aa₃* complex.

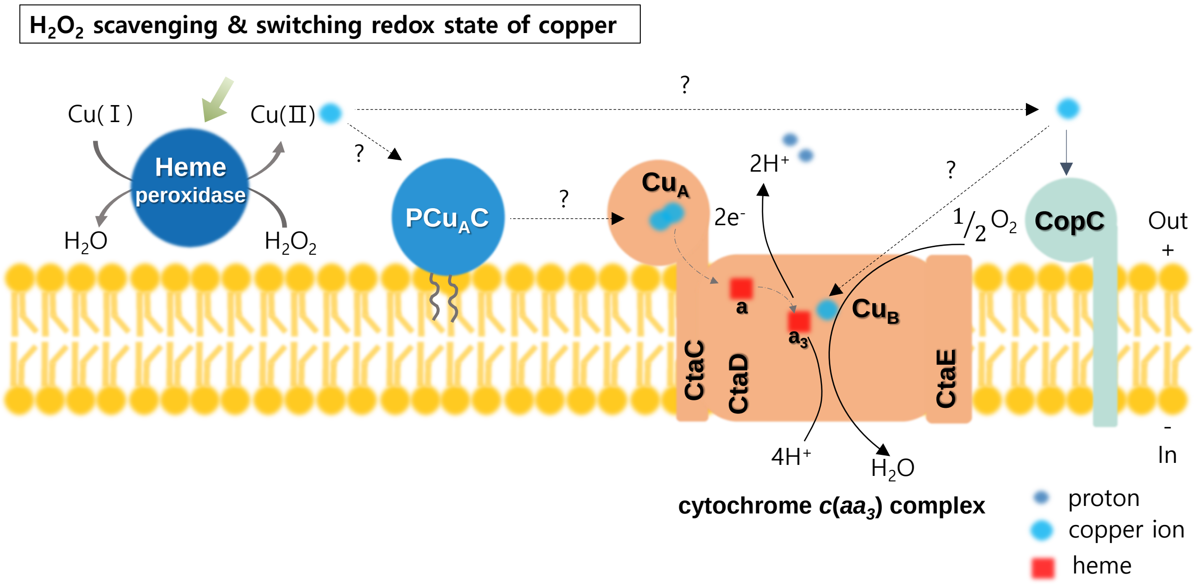

The NCgl1608, NCgl1609, NCgl1610 were represented as heme peroxidase, PCu_AC, and CopC, respectively. PCu_AC is a putative membrane-associated lipoprotein, and CopC is a putative copper importer. Both proteins have copper-binding motifs and might contribute to copper incorporation in cytochrome c oxidase. The dyp-type heme peroxidase secreted via the TAT system is assumed to switch the redox state of copper ion in media and facilitate the metalation of cytochrome *c* oxidase while scavenging H_2O_2.

(L 253)

Figure S3. (A) SWISS-modelling and (B) CD-search of NCgl2435, a hypothetical peptidyl-tRNA hydrolase.

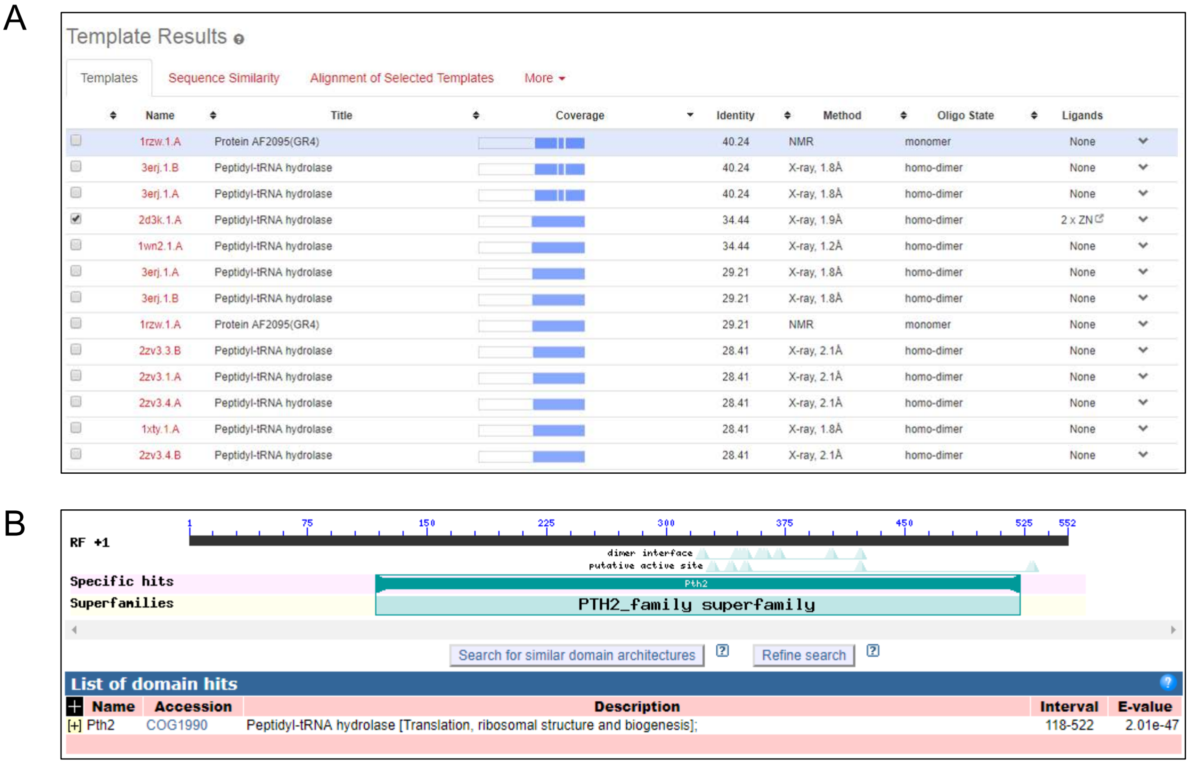

A

Template Results

Templates | Sequence Similarity | Alignment of Selected Templates | More

	Name	Title	Coverage	Identity	Method	Oligo State	Ligands
☐	1rzw.1.A	Protein AF2095(GR4)		40.24	NMR	monomer	None
☐	3erj.1.B	Peptidyl-tRNA hydrolase		40.24	X-ray, 1.8Å	homo-dimer	None
☐	3erj.1.A	Peptidyl-tRNA hydrolase		40.24	X-ray, 1.8Å	homo-dimer	None
☑	2d3k.1.A	Peptidyl-tRNA hydrolase		34.44	X-ray, 1.9Å	homo-dimer	2 x ZN
☐	1wn2.1.A	Peptidyl-tRNA hydrolase		34.44	X-ray, 1.2Å	homo-dimer	None
☐	3erj.1.A	Peptidyl-tRNA hydrolase		29.21	X-ray, 1.8Å	homo-dimer	None
☐	3erj.1.B	Peptidyl-tRNA hydrolase		29.21	X-ray, 1.8Å	homo-dimer	None
☐	1rzw.1.A	Protein AF2095(GR4)		29.21	NMR	monomer	None
☐	2zv3.3.B	Peptidyl-tRNA hydrolase		28.41	X-ray, 2.1Å	homo-dimer	None
☐	2zv3.1.A	Peptidyl-tRNA hydrolase		28.41	X-ray, 2.1Å	homo-dimer	None
☐	2zv3.4.A	Peptidyl-tRNA hydrolase		28.41	X-ray, 2.1Å	homo-dimer	None
☐	1xty.1.A	Peptidyl-tRNA hydrolase		28.41	X-ray, 1.8Å	homo-dimer	None
☐	2zv3.4.B	Peptidyl-tRNA hydrolase		28.41	X-ray, 2.1Å	homo-dimer	None

List of domain hits

	Name	Accession	Description	Interval	E-value
[+]	Pth2	COG1990	Peptidyl-tRNA hydrolase [Translation, ribosomal structure and biogenesis];	118-522	2.01e-47

(L 254)

Table S2. Relative transcriptional levels of genes related to leucine, isoleucine, valine biosynthesis.

NCgl No.	Gene name	Function of gene product	mRNA ratio[a)]
NCgl1222	*ilvB*	Acetolactate synthase	1.26
NCgl1223	*ilvN*	Acetohydroxyacid synthase small subunit	1.67
NCgl1224	*ilvC*	Ketol-acid reductoisomerase	1.24
NCgl1219	*ilvD*	Dihydroxy-acid dehydratase	1.71
NCgl2123	*ilvE*	Branched-chain amino acid aminotransferase	1.14
NCgl0245	*leuA*	2-Isopropylmalate synthase	1.05
NCgl1262	*leuC*	3-Isopropylmalate dehydratase large subunit	8.53
NCgl1263	*leuD*	3-Isopropylmalate dehydratase small subunit	7.05
NCgl1237	*leuB*	3-Isopropylmalate dehydrogenase	2.24

[a)] mRNA ratio for JH41/PT
